# Staff Attitudes Toward Healthcare Waste Separation: An Exploratory Survey from a Triple-Bottom-Line Perspective

**DOI:** 10.3390/healthcare14080975

**Published:** 2026-04-08

**Authors:** Julia Nike Sturm, Mark Berneburg, Bernadett Kurz, Dennis Niebel

**Affiliations:** 1 Department of Dermatology, University Medical Center Regensburg, Franz-Josef-Strauss Allee 11, 93053 Regensburg, Germany; mark.berneburg@klinik.uni-regensburg.de (M.B.); bernadett.kurz@klinik.uni-regensburg.de (B.K.); dennis.niebel@dermpath-koelnbonn.de (D.N.); 2 Heinz-Werner-Seifert-Institut, Trierer Str. 70-72, 53115 Bonn, Germany

**Keywords:** sustainable clinical care, waste management, dermatology, planetary health, resource efficiency

## Abstract

Background: In 2022, the German healthcare system generated 400,000 tons of waste. Reducing this number could lower greenhouse gas emissions. The waste management plan at the University Medical Center Regensburg, and those of other comparable German facilities, require that glass, cardboard/paper, residual waste, and other non-hazardous materials are collected separately. Objectives: To assess the personal interest, proficiency, opinion, and awareness of waste management among German dermatology staff to develop customized, resource-saving process optimization and training programs. Methods: An online cross-sectional survey was conducted among German dermatology healthcare professionals between 27 February and 4 October 2024. Out of the 100 responses, 84 were complete and subsequently analyzed. Respondents included staff at dermatology wards, outpatient units, and private practices. Data were analyzed descriptively; comparisons were made between clinics and outpatient units, and correlations were identified among the items. Results: Most respondents perceived the amount of waste generated during wound dressing changes as high; more than 60% expressed an interest in receiving further training on sustainability and waste reduction. Although many respondents reported having a good understanding of waste separation, they identified time pressure and stress as the two main obstacles to consistent implementation. Higher self-reported knowledge did not correspond with greater confidence in recycling as an effective waste reduction measure. Conclusions: The findings suggest a discrepancy between awareness and practice regarding sustainable waste management in dermatology. Combining structural and organizational measures with targeted training and workflow optimization could promote more sustainable clinical practices.

## 1. Introduction

Between 2014 and 2023, the average annual generation of waste in Germany amounted to approximately 407 million tons. Despite the federal waste prevention program adopted in line with the EU requirements in 2012 [1,2], the volume of waste showed no substantial change over that period ([3] own calculation).

The Circular Economy Act (CEA), which describes the waste hierarchy as a guideline, was passed to protect raw materials and useful goods. The first step is to avoid generating waste, followed by direct reuse or implementing a recycling process. Disposal is only a last measure [4]. In the healthcare system, however, the CEA should be implemented not only to conserve raw materials, but also to protect everyone involved with ‘hazardous’ waste.

According to the World Health Organization (WHO), 85% of the waste generated in the healthcare system is classified as ‘general’ or ‘non-hazardous’ [5]. This refers to waste that does not pose a biological, chemical, or radioactive risk and includes materials similar to regular municipal waste, such as packaging, paper, and food waste [6] (p. 3). In Germany, 430,300 tons were generated in the European Waste Catalogue (EWC) 18 in 2022, which includes ‘waste from human medical or veterinary care and research’. Of this, 400,300 tons were ‘waste, whose collection and disposal is not subject to special requirements from an infection prevention perspective’ (EWC 180104 and 180203), which equals normal household waste. This category therefore amounted to an above-average 93%, compared to the WHO’s estimated general share of 85% ([7] own calculation). On average, the healthcare sector contributes 4.4% to total national CO_2_ emissions worldwide [8].

Emissions are divided into three scopes: Scope 1 covers direct emissions from owned sources; Scope 2 includes indirect emissions from purchased energy; and Scope 3 represents indirect emissions from the entire value chain, such as the production and transport of disposables, equipment, pharmaceuticals, and waste management. Scope 3 typically accounts for 50–75% of total emissions, making it the largest and most challenging category to reduce [9]. This is why it is important to respect the waste hierarchy as outlined in the CEA to improve the carbon footprint of healthcare.

The total emissions over a one-year assessment period in a high-volume outpatient dermatology department were approximately 323.6 tons of CO_2_-equivalent (tCO_2_e). The largest share stemmed from Scope 3 emissions (51.1%), notably from purchased goods and services (e.g., medical materials), patient travel, and waste, which contributed 13.1 tCO_2_e or 7.9% of Scope 3 emissions. Scope 2 emissions from steam and chilled water made up 46.3%, and Scope 1 (e.g., heating) only 2.5% [10].

Dermatology and other surgical specialties are resource-intensive due to the frequency of procedures and the resulting waste, particularly of sterile, single-use materials. Dermatology ranks just below other surgical fields in terms of equipment use, energy consumption, and waste generation, and produces significantly more waste than non-procedural specialties [11]. Dermatology also generates waste due to the widespread distribution of product samples with an unfavorable CO_2_/water-use ratio that often come in non-recyclable packaging [12]. In procedure-oriented and time-sensitive specialties, the translation of sustainability goals into routine clinical practice therefore depends not only on knowledge but also on the practical feasibility within existing workflows [13].

The healthcare sector is significantly affected by the consequences of climate change, for instance, an increased incidence of heat stroke and the aggravation of chronic conditions, such as cardiovascular and respiratory diseases [14] (p. 64). At the same time, the healthcare sector contributes substantially to greenhouse gas emissions [9]. To lower this environmental impact, it is essential to work resource-efficiently, reduce waste, and optimize recycling. The successful implementation of sustainable practices in healthcare is strongly influenced by structural and organizational conditions, including workflow integration, time constraints, and process-related constraints [13]. The Triple-Bottom-Line definition of sustainability emphasizes that these measures should consider ecological, social, and economic aspects. This way, sustainable practices help achieve a balance between environmental protection, social responsibility, and economic factors [15]. Through informed purchasing decisions and appropriate waste management, healthcare staff can help reduce emissions and environmental impact. This challenge reflects what has been described as an awareness–behavior gap, where positive environmental attitudes and intentions do not automatically result in consistent pro-environmental behavior. Given the limited data on this topic, this exploratory study aimed to answer the following research questions: (1) How do staff perceive the importance and practicability of sustainable waste management in dermatology? (2) What is the relationship between the perceived potential for waste reduction and its actual implementation in practice? (3) How is increased recycling evaluated as a waste reduction measure? The study also aimed to identify opportunities for training, awareness programs, and capacity building to promote sustainable practices.

## 2. Materials and Methods

### 2.1. Study Design, Setting, and Participants

From 27 February 2024 to 4 October 2024, a prospective, cross-sectional, online-based survey was conducted among dermatology healthcare professionals working in outpatient practices in Germany. The source population included healthcare professionals in dermatological hospitals and outpatient settings: doctors, nurses, medical students, medical assistants, and technical assistants.

A convenience sampling approach was used because no prior literature or reference data were available for a formal sample size calculation.

The survey was distributed via a mailing list to the employees of the Department of Dermatology at the University Medical Center Regensburg (111 persons eligible to participate), and 31 university hospitals were asked to forward it accordingly. In this process, the secretaries of the chief physicians at each hospital were directly contacted to facilitate distribution. Three university hospitals confirmed forwarding, reaching approximately 300 university-based healthcare professionals.

Healthcare professionals in the outpatient setting (OP-HCPs) across 23 dermatology practices in Bavaria were contacted using publicly available online information. Since most of the practices were located in Bavaria, geographic bias may limit generalizability, and not all practices may have been reached.

Participants were also approached at two dermatology-focused professional conferences: the ‘KoPra-kompakt + praxisnah 2024’ in Wiesbaden, a three-day dermatology congress attended by approximately 1400 healthcare professionals, and the ‘44th Erlanger Minisymposium’ on dermatological practice and research, where a total of 300 QR codes were distributed. Approximately 940 persons were reached (≈410 from 4 university dermatology departments, ≈230 from 23 practices, and 300 via QR codes at conferences). A total of 84 complete surveys were received, resulting in an estimated response rate of approximately 9%.

### 2.2. Variables and Measurement

Data were collected via an online survey using SoSciSurvey Version 3.4.14 (Munich, Germany), ensuring data protection through collaboration with the University of Regensburg. Two versions of the survey were developed: one tailored for healthcare professionals in an inpatient dermatology ward (IDW-HCPs), and the other for OP-HCPs. Detailed participant and data protection information were provided. Participation required informed consent, ensured by a mandatory checkbox.

The independent variables included sociodemographic factors (sex, age, and educational/profession) and the distinction between IDW-HCP and OP-HCP. The sex categories were male, female, or diverse. Age was recorded in 10-year age categories. IDW-HCPs were classified as doctors and nurses. OP-HCPs included doctors and medical assistants. There was also the option “other”.

**Figure 1:**
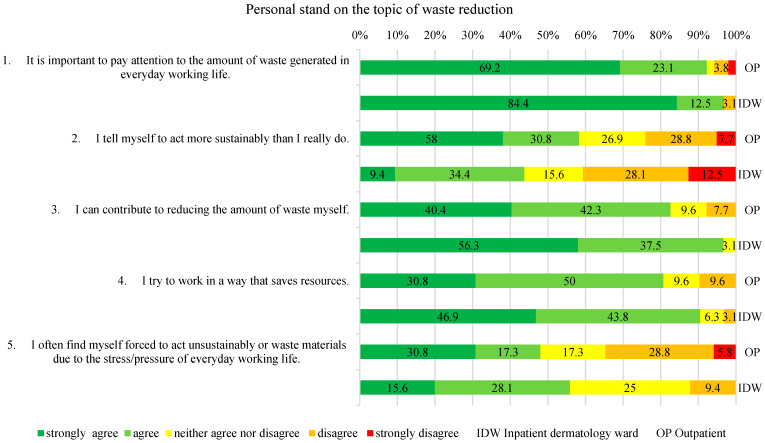
Personal stand on the topic of waste reduction.

**Figure 2:**
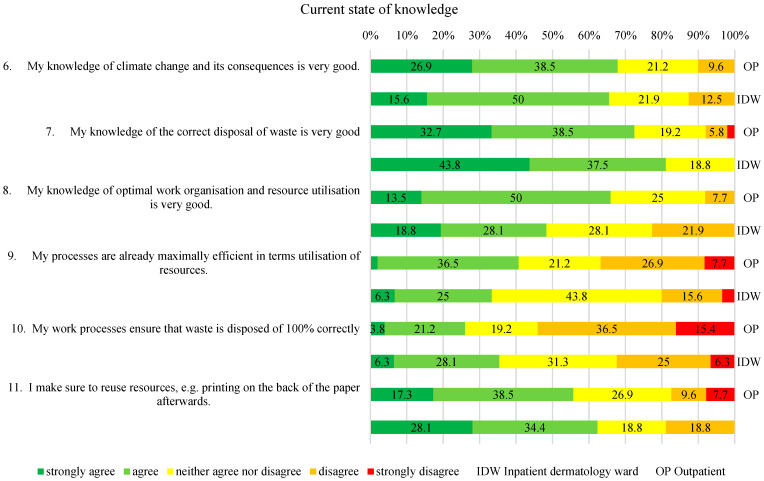
Current state of knowledge.

**Figure 3:**
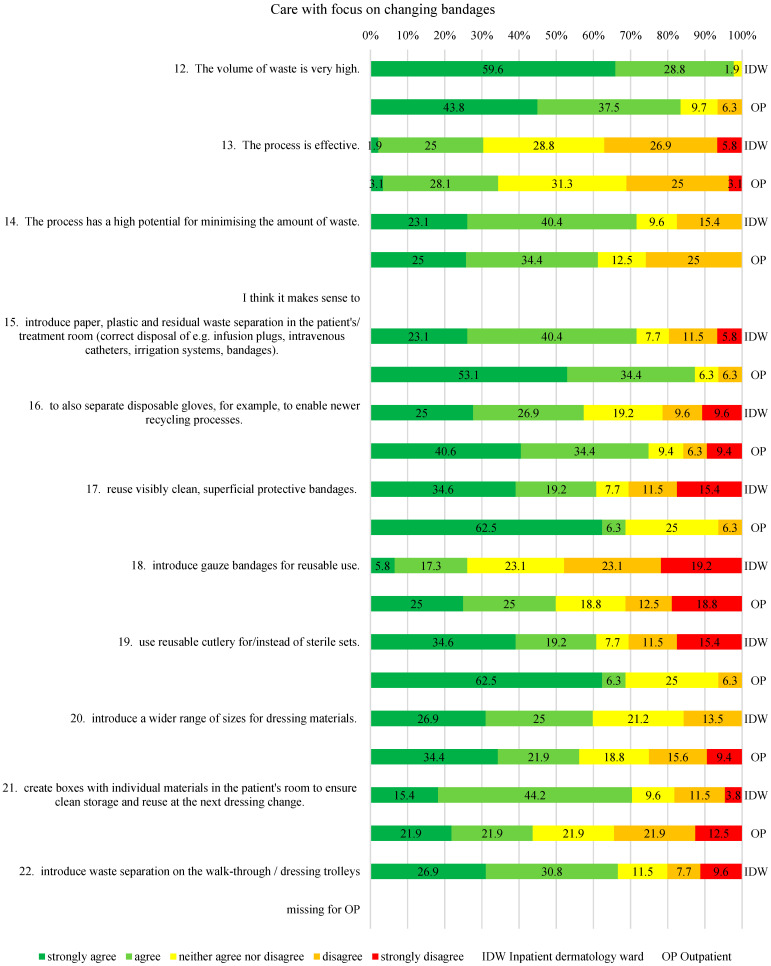
Care with focus on changing bandages.

**Figure 4:**
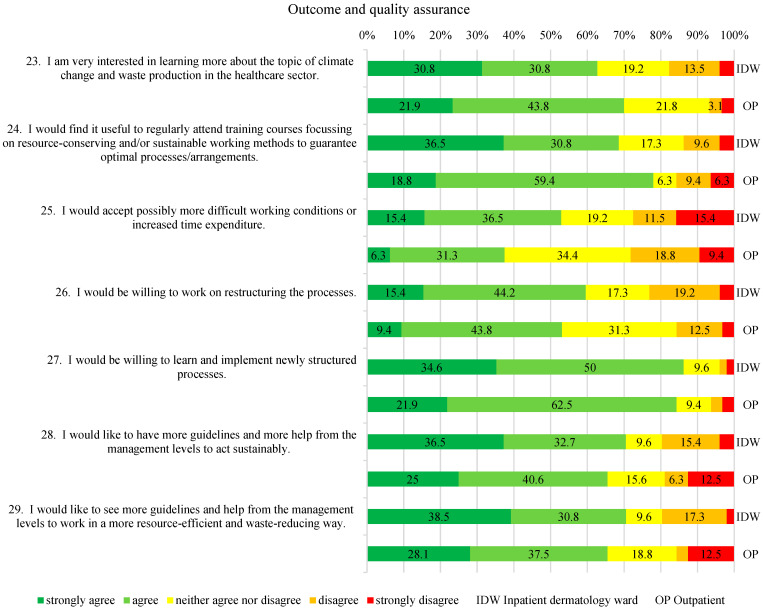
Outcome and quality assurance.

The dependent variables were measured using a study-specific questionnaire with 5-point Likert scales (strongly agree to strongly disagree), consistent with established Knowledge–Attitudes–Practices (KAP) survey methodology widely used in health services research to explore perceptions and self-reported behavior [16]. The questionnaire included four domains. These domains were defined a priori to correspond to the study’s research questions and aim: Theme 1 (“Personal stance on waste reduction”) addressed Research Questions 1 and 2 regarding awareness and perceived importance; Theme 2 (“Current knowledge”) informed Research Questions 1–3, providing insights into awareness, practicability, and potential for recycling; Theme 3 (“Care with a focus on changing bandages”) focused on Research Question 3 while also exploring additional waste-reduction measures, which were compared to inform the study aim of identifying staff training and awareness opportunities; and Theme 4 (“Outcome and Quality Assurance”), together with Theme 3, contributed directly to the study aim by highlighting areas for education, capacity building, and process optimization. As no validated instrument was available for this clinical context, a study-specific questionnaire was developed following a targeted PubMed search confirming the absence of comparable instruments in dermatology or related settings. Because the study relied exclusively on self-reported data, responses reflect declared attitudes and practices rather than objectively measured behavior, which may be subject to reporting bias or social desirability. Misunderstandings of individual questions cannot be entirely ruled out, particularly in the absence of interviewer guidance. The complete survey statements are presented in Figure 1, Figure 2, Figure 3 and Figure 4.

### 2.3. Statistical Analysis

No suitable data were available for a power analysis. Therefore, a target sample size of 100 participants was set for the pilot survey, as this number was considered feasible within the practical constraints of the study. Of the 101 HCPs who participated in the study, 17 were excluded due to missing data, leaving 84 valid questionnaires for further analysis. The relatively small final sample size limits statistical power and restricts the detection of small or moderate effects. The data were processed using IBM SPSS Statistics Version 29.0.0.0 (Armonk, NY, USA). These were first divided into two groups: one with IDW-HCPs and the one with OP-HCPs. Equivalent statements on ‘Care with a focus on dressing changes’ were adjusted for comparability. Frequency tables were created for all items. Because the items addressed different aspects and the study was exploratory, the items were analyzed individually rather than being combined into scales. To detect differences between groups, the Mann–Whitney U test was applied. In addition, item-level correlation analyses were conducted to provide an overview of relationships between statements, with reporting limited to correlations relevant to the research objectives. Due to the small sample size and non-normal data, more complex inferential analyses, such as ANOVA, were not feasible.

## 3. Results

### 3.1. Socio-Demographic Details of the Participants

In total, there were 18 male and 66 female participants. Among the 52 IDW-HCPs, most were doctors (19) or nurses (14), reflecting a similar distribution to the group of OP-HCPs that included 15 doctors and 8 medical assistants. The 26–35 age group was represented the most often, with 26 participants; the remaining age groups were largely homogeneous (5–9). These details are also presented in Table 1.

### 3.2. Overview of Response Frequencies

The section “Personal stand on the topic of waste reduction” shows that most respondents from both groups considered resource-saving work important, achievable, and worth implementing (see Figure 1, Nos. 1, 3, 4). In both groups, there was predominant indecision or a slight tendency toward agreeing or disagreeing when perceiving one’s own behavior as more sustainable than it actually was (see Figure 1, No. 2). IDW-HCPs more frequently reported being forced into resource-wasting behavior due to lack of time and perceived stress (48.1% agreeing or somewhat agreeing) than OP-HCPs (53.1% somewhat agreeing or undecided; see Figure 1, No. 5). There were no significant differences between the groups for this question or any other question in this section (Mann–Whitney U test, each *p* > 0.05).

In the section “Current knowledge”, more than 50% of respondents from both groups reported having a good understanding of climate change and proper waste separation (see Figure 2, Nos. 6, 7). Regarding optimal work processes and resource utilization, more than 50% of respondents from both groups were undecided or somewhat agreed (see Figure 2, No. 8). A total of 55.8% of IDW-HCPs and 62.5% OP-HCPs were undecided or rated the effectiveness of their work processes negatively (see Figure 2, No. 9), while 56% of IDW-HCPs and 56.3% of OP-HCPs considered proper waste separation to be “rather not possible” or “indeterminable” (see Figure 2, No. 10). Most respondents reported being mindful of double-sided printing (see Figure 2, No. 11). There were no statistically significant differences between groups for this question and any other question in this section (Mann–Whitney U test, each *p* > 0.05).

In the “Care with a focus on changing bandages” section, 88.4% of IDW-HCPs and 81.3% of OP-HCPs agreed that the amount of waste in this category was very high (see Figure 3, No. 12), with 63.5% of IDW-HCPs and 59.4% of OP-HCPs seeing a high potential for waste reduction (see Figure 3, No. 14). The majority were undecided (or with a slight tendency in one direction) about whether the implemented process was already the most efficient one. Most respondents from both groups rated waste reduction measures in the “care with a focus on dressing changes” section as sensible (see Figure 3, Nos. 15, 16, 20, 21, 22). Significant differences were observed for specific suggestions (Mann–Whitney U test, *p* < 0.05): 62.5% of OP-HCPs supported the reuse of clean-appearing dressings (see Figure 3, No. 17; *p* = 0.007), while IDW-HCPs showed more varied opinions. A similar pattern appeared regarding the introduction of reusable surgical instruments (see Figure 3, No. 19; *p* = 0.03). Reusable gauze bandages were rejected by 42.3% of IDW-HCPs, while 50% of OP-HCPs agreed (see Figure 3, No. 18; *p* = 0.049).

In the “Outcome and Quality Assurance” section, more than 60% of respondents from both groups expressed interest in further training on resource-saving work and climate change in healthcare (see Figure 4, Nos. 23 and 24). Most participants also supported receiving more guidelines from supervisors regarding sustainability and waste reduction (see Figure 4, Nos. 28, 29). A total of 59.6% of IDW-HCPs and 53.2% of OP-HCPs (see Figure 4, No. 26) agreed with the development of new processes, while the willingness to accept more time-consuming or more difficult working conditions was lower (51.9% IDW-HCPs; 37.6% OP-HCPs; see Figure 4, No. 25). More than 75% of the participants were willing to learn new processes (84.6% IDW-HCPs; 84.4% OP-HCPs).

### 3.3. Key Correlations in Sustainability Practices

All items of the self-developed questionnaire were correlated to examine relationships between the assessed aspects of sustainable waste management. The results are presented as a correlation matrix in Table 2 (Key correlations) and are structured according to the research questions. The analysis particularly focused on correlations related to the perceived importance of sustainability, the perceived potential for waste reduction in relation to its practical implementation in everyday clinical work, and the evaluation of recycling and waste separation measures. In addition, correlations indicating potential for further sustainable development of the clinical working environment are reported.

Initially, a positive correlation was observed between Statement 1 and Statement 3, This indicates that participants who consider sustainable actions to be important are more likely to believe that they can personally contribute to reducing waste.

Building on this, a positive correlation coefficient indicates that higher levels of knowledge and a better assessment of one’s own work processes (Nos. 8 and 9) are associated with a more consistent implementation of resource-saving behavior (No. 4). Participants who perceive their knowledge and work processes as good report being less often forced to act unsustainably due to stress, reflecting a negative correlation between knowledge/process efficiency (No. 8, 9) and stress (No. 5).

At the same time, a negative correlation coefficient exists between Statement 2 (intentional sustainable behavior) and Statement 8 (knowledge of process optimization and resource use). As knowledge decreases, participants are more likely to overstate their intentional sustainable behavior. This behavior also correlates positively with perceived stress (Nos. 2 and 5) and negatively with the perceived ability to personally reduce waste (No. 3): the more participants report neglecting sustainable practices due to stress, the more they claim to act sustainably and the less they believe they can reduce waste.

Another positive correlation was observed between the perception of high waste volumes (No. 12) and stress related to sustainable practices (No. 5). Similarly, stress positively correlates with the desire for alternative materials, such as different bandage sizes (No. 20): the more participants felt affected by stress, the more they perceived waste levels as high and expressed a need for a wider range of materials, such as bandages, to reduce waste.

Conversely, with increasing knowledge or greater work process efficiency (Nos. 8 and 9), the likelihood of viewing waste separation as an optimization opportunity decreases, as indicated by a negative correlation with Statement 15. At the same time, Statement 15 shows positive correlations with Statement 1, reflecting greater attention to sustainability, and with Statement 2, indicating higher self-reported intentional sustainable behavior.

Finally, a positive correlation coefficient indicates that concerns about waste generation and greater knowledge of climate change (Nos. 1 and 3) are associated with a stronger desire for guidelines and greater willingness to adopt new processes (Nos. 28 and 29).

## 4. Discussion

### 4.1. Summary of Main Findings

Our findings suggest that although sustainable waste management in dermatological practice is widely regarded as important, it remains challenging to implement consistently in daily clinical routines. A central observation of this exploratory study is the gap between high awareness and limited practicability, which may indicate that sustainability may be constrained less by a lack of knowledge than by social, organizational, and economic barriers.

Although staff perceived considerable potential for waste reduction, this potential appears only partially realized in practice, as time pressure, stress, and workflow complexity were described as obstacles to correct waste separation. In addition, increased recycling was evaluated ambivalently: while its ecological relevance was acknowledged, more knowledgeable staff were more likely to report questioning its effectiveness when not supported by optimized processes.

Taken together, these descriptive findings point to a potential Triple-Bottom-Line challenge: ecological goals are accepted, but perceived social and economic constraints may limit feasibility, suggesting that structural and organizational factors may warrant further investigation.

Because the analyses are exploratory, the results should be interpreted descriptively and as hypothesis-generating rather than as confirmatory evidence.

### 4.2. Interpretation and Comparison with Literature

Regarding the first research question, the present findings provide initial insights into how dermatology staff perceive both the importance and the practicability of sustainable waste management. Consistent with previous research [17,18], the majority of respondents rated sustainable waste separation as important, which may reflect a high level of environmental awareness among healthcare professionals (Figure 1, No. 1). From a Triple-Bottom-Line perspective, recycling systems are widely recognized as ecologically beneficial due to their potential to reduce greenhouse gas emissions and are economically viable through resource conservation—provided that waste separation is performed accurately. However, perceptions of practicability appear more ambivalent. Staff members who report experiencing high levels of work-related stress and feeling compelled to deprioritize sustainable behavior are statistically more likely to report themselves as generating excessive amounts of waste and to acknowledge a tendency to overestimate their own sustainable practices (positive correlations between Nos. 2 and 12 with No. 5; see Table 2) Conversely, participants with higher knowledge and more efficient work processes reported experiencing less stress related to sustainable practices, reflecting negative correlations between Nos. 8 and 9 with No. 5 (see Table 2). This pattern may suggest that while the importance of sustainable waste management is broadly acknowledged, its consistent implementation is perceived as challenging under everyday clinical conditions. This observation is consistent with discussions in implementation science and planetary, which emphasizes that the sustainability of healthcare interventions depends largely on organizational, contextual, and system-level factors [19]. In addition, perceived self-efficacy may be associated with sustainability attitudes: participants who attach greater importance to sustainability are more likely to believe that they can personally contribute to waste reduction (positive correlation between Nos. 1 and 3; see Table 2). These associations should be interpreted cautiously and as exploratory observations rather than evidence of causal effects, as the limited sample size and correlational approach restrict inferential strength.

Addressing the second research question on the relationship between the perceived potential for waste reduction and its actual practical implementation, the present findings suggest the presence of what may be described as an implementation gap rather than a lack of knowledge. While more than half of the respondents from both groups reported feeling well-informed about waste separation (Figure 2, No. 7), a similar proportion doubted or remained uncertain about its feasibility (Figure 2, No. 10). Although self-selection bias cannot be excluded due to the voluntary nature of the study and the low response rate, this contrast suggests that limited implementation may be associated more with structural and organizational constraints than with informational deficits. Concerns about waste accumulation and disposal were particularly pronounced among nursing trainees with recent long-term placements [20] (p. 4). Even in a potentially sustainability-oriented sample, time pressure and stress were described as relevant social factors impeding sustainable practices (Figure 1, No. 5), which the respondents described as making fully correct waste separation difficult to achieve under routine working conditions. This observation is comparable to findings reported in previous studies that reported logistical challenges and confusion in assigning the correct waste types to the appropriate color-coded bins [21]. Taken together, the results may point toward the relevance of the social pillar of the Triple-Bottom-Line, as sustainable behavior appears to be shaped more by workload, workflow complexity, and perceived feasibility than by knowledge alone. Because the study is exploratory and based on voluntary participation, these barriers may be over- or underrepresented and are not generalizable, but they still provide valuable insights into social and organizational challenges and generate hypotheses for further research.

Building on the identified social and organizational constraints, the third research question shifts the focus from general implementation barriers to the evaluation of recycling as a specific waste reduction strategy. In this context, institutional insights from the hygiene officer at the University Medical Center Regensburg indicate that incorrect waste separation increases disposal costs, as misclassified waste must be treated as hazardous and disposed of accordingly, thereby undermining the economic pillar of sustainability and contributing to the perception that full compliance is difficult to achieve in practice. The limited feasibility of consistent waste separation may reflect a key Triple- Bottom-Line challenge: although ecological objectives are widely acknowledged, persistent social and economic constraints may limit the perceived feasibility of predominantly individual-focused approaches. Against this background, correlation analyses provide an exploratory perspective on how recycling is perceived in everyday practice, given that the cross-sectional design is limited to associations at a single point in time and does not permit causal or long-term inferences. The correlations suggest an association in which waste separation is perceived as an optimization measure mainly among participants with high sustainability awareness or among those who reported that they may overestimate their own sustainable practices (positive correlations between Nos. 1 and 2 with 15; see Table 2). In contrast, participants with higher levels of knowledge and more efficient work processes appear less likely to perceive waste separation as a meaningful optimization opportunity (negative correlations between Nos. 8 and 9 with No. 15; see Table 2). This pattern may indicate that within this sample, recycling is sometimes perceived as a low-threshold or symbolic sustainability measure rather than as a central component of comprehensive process optimization. From an organizational perspective, these exploratory associations may raise the question of whether time-consuming and logistically complex recycling measures require coordinated guidance, standardized procedures, and streamlined workflows in order to be implemented effectively. Prior research has similarly indicted that structural and contextual conditions are often more strongly associated with sustainable behavior than individual motivation alone [22].

### 4.3. Practical Implications

In conclusion, the results suggest that within this sample and interpreted through a Triple-Bottom-Line framework, increased waste separation alone was perceived as having potentially limited impact in this setting. Ecological benefits can be realized when social and economic constraints are addressed, which highlights the need for structural and organizational improvements rather than relying on individual behavior. Sustainable transformation in healthcare may involve context-specific, exploratory measures that integrate staff knowledge, process efficiency, and capacity building.

According to the WHO’s 10 components of climate-resilient health systems, staff training (component 2) and both resource efficiency and adaptability (component 3) are essential pillars of sustainable transformation [23]. This is supported by the data: environmentally friendly behavior correlates with both high knowledge and optimized workflows (positive correlations between Nos. 8 and 9 with No. 4; see Table 2). Furthermore, more than 60% of the respondents from both groups expressed an interest in sustainability training (Figure 4, Nos. 23 and 24). In line with these two components, the dermatology staff expressed a strong interest in clear guidelines and in learning new processes to improve adaptability and resource efficiency, particularly among those with higher climate change awareness (positive correlation between Nos. 28 and 29 with Nos. 1 and 3, see Table 2). The exploratory correlations reported under Research Question 3 suggest that participants with higher knowledge and more efficient workflows were less likely to perceive recycling as a meaningful optimization opportunity. This pattern may indicate that within this specific sample, continuing education in dermatology could explore process optimization rather than introducing additional recycling measures. Such an approach might align training with practical opportunities to improve resource efficiency within existing structural constraints. These preliminary and exploratory observations suggest possible directions for practice, but they pertain only to this specific sample and should be interpreted with caution; they also underscore the importance of combining subjective survey data with objective process indicators in future research to strengthen the evidence base.

Potential low-threshold measures discussed in relation to the observed associations include introducing a more differentiated range of dressing material sizes (positive correlation between No. 5 and No. 20, see Table 2) and optimizing waste bin placement. Previous research has reported that the close proximity of hazardous and non-hazardous bins may be associated with improved waste separation outcomes [24]. In the present exploratory context, such measures may represent areas for further investigation regarding process optimization. However, these suggestions are based on exploratory cross-sectional data and require validation in larger, more diverse settings. Taken together, the results descriptively identify potential areas in which educational or capacity-building initiatives could be explored in dermatology, particularly in relation to the association between knowledge, workflow organization, and self-reported sustainable practices.

The video (see Supplementary Material) was developed as an illustrative example of process optimization concepts using a structured Plan Do Check Act (PDCA) model [25]. It begins by presenting the importance of sustainability and the need to reduce waste in dermatology and healthcare, based on the current literature, and outlines steps for planning, implementing, monitoring, and adjusting workflow improvements related to waste management and sorting. The video does not include additional recycling measures as recycling initiatives that were considered potentially complex and time-consuming for staff. Existing recycling practices were not replaced. It should be noted that the video and the model represent a first attempt and a practical opportunity to assess their usefulness and impact in real-world settings, rather than a definitive solution, and their effectiveness should be further evaluated in future studies. The video is freely accessible on the German Dermatological Society’s Sustainability Working Group homepage and is also publicly available on Zenodo.

### 4.4. Limitations

This study has several limitations. First, it should be emphasized that this is a pilot study and was not intended to provide statistically representative or generalizable results. The sampling strategy represents a key limitation: the use of convenience sampling, combined with limited institutional participation and voluntary online recruitment, may have introduced selection bias and self-selection effects.

As a result, the sample may overrepresent younger, more highly educated, or sustainability-oriented participants. In addition, the relatively low response rate raises the possibility of non-response bias, meaning that participants may systematically differ from non-participants in relevant characteristics, such as motivation or interest in sustainability.

Furthermore, the small sample size limits statistical power and restricts the detection of smaller effects. Another important limitation is the reliance on self-reported data, as no objective behavioral measures were included. Consequently, responses reflect declared attitudes and practices rather than actual behavior, which may reduce accuracy.

A specific limitation is social-desirability bias, where respondents may overreport socially favorable attitudes or practices. Social-desirability bias is a common threat to the validity of self-report measures in health research and can lead to systematic distortion of survey results [26]. No specific measures were implemented in this study to assess or control socially desirable responding.

In addition, the use of a study-specific, non-validated questionnaire may limit construct validity and comparability with other studies. Misunderstandings of individual questions cannot be entirely ruled out, particularly in the absence of cognitive pretesting or interviewer guidance.

Finally, the cross-sectional design of the study does not allow for conclusions regarding causal relationships or the long-term effects of individual measures.

### 4.5. Further Research

Given the exploratory nature of the study, voluntary participation, and the limited sample size, the findings cannot be generalized. However, they provide several methodological lessons for future research.

Future studies should aim to recruit broader and more representative samples, for example, stratifying participants by region, institution type, and professional group, and by collaborating with professional associations to reach a wider population. This could be achieved by applying stratified sampling strategies, such as cluster sampling, instead of convenience sampling, supported by a clearer sampling frame (e.g., professional registries or institutional databases). Underrepresented groups, including older staff or those less sustainability-oriented, should be actively recruited to enhance representativeness. Standardized follow-up strategies such as reminders or participation incentives may help to improve response rates and reduce non-response bias. Comparisons between respondents and non-respondents could help assess potential bias by using available demographic or institutional data.

Survey instruments should be refined through cognitive pretesting or pilot testing to ensure clarity and minimize the risk of misinterpretation and expert review, or formal validation should be performed to ensure construct validity and comparability with other studies [27]. To reduce socially desirable responding, validated social-desirability scales (e.g., short forms of the Marlowe–Crowne Social Desirability Scale) can be incorporated to detect or control for socially desirable responding in analyses [28], or by applying indirect questioning techniques, such as the randomized response method, which have been shown to reduce socially desirable responding in epidemiologic surveys compared with direct questions [29]. In addition, items should be behaviorally anchored, neutrally worded, and focused on observable actions rather than attitudes, using time-bound reference frames to further minimize response bias. Combining self-reports with objective behavioral measures (e.g., observational assessments of actual waste separation) might further strengthen validity.

Statistical conclusion validity should be supported by multivariable regression models with pre-specified covariates, including demographic and institutional factors as well as social desirability scores. Sensitivity analyses should test whether results remain stable after excluding participants with high social desirability scores and using alternative operationalizations of key variables. In addition, robustness checks should include subgroup analyses (e.g., by profession, institution type, and region) and the assessment of interaction effects. Missing data should be examined, and sensitivity analyses (e.g., complete-case vs. imputed data) should be performed to evaluate potential bias.

Future studies should investigate staff knowledge of structured process optimization approaches and their implementation in routine clinical practice. It is also important to examine whether such approaches are associated with measurable improvements in waste management using objective indicators and longitudinal or controlled study designs. In addition, educational interventions that incorporate structured workflow models (e.g., the PDCA model, as introduced in the educational video [25]) should be evaluated using methodologically robust designs, including controlled or longitudinal studies that combine self-reported and objective behavioral measures.

The present study does not allow for conclusions about the effectiveness of process optimization strategies, training programs, or educational tools. Taken together, the findings generate hypotheses about how knowledge, perceived feasibility, and workflow organization may interact in shaping sustainable practices in dermatological settings and are intended to inform future research rather than to demonstrate the efficacy of specific interventions.

## 5. Conclusions

This pilot study suggests the presence of a gap between awareness and the practical implementation of sustainable waste management within a specific dermatological clinical setting. Although staff reported substantial knowledge and motivation regarding sustainability, structural and organizational barriers, such as time pressure, stress, and logistical challenges, appeared to limit consistent waste sorting and recycling practices. The findings suggest that in this sample, sustainability in daily clinical work may be shaped more strongly by workflows and institutional conditions than by individual knowledge alone.

As an exploratory investigation conducted in a single disciplinary and institutional context, these results should be interpreted with caution. The findings are context-dependent and primarily hypothesis-generating, limiting their transferability to other specialties, institutions, or healthcare systems.

Based on these exploratory insights within this specific sample, practical considerations can be cautiously outlined. The observed interest in continuing education may indicate that in this particular context, training initiatives could explore opportunities for process optimization within existing structural constraints, rather than focusing solely on additional recycling measures. However, these preliminary observations do not permit definitive conclusions regarding effectiveness. Further research in larger and more diverse samples is required to test context-specific interventions, evaluate structural and organizational determinants longitudinally, and assess outcomes using both self-reported and objective behavioral measures before broader or transferable recommendations can be made.

## Figures and Tables

**Table 1:** Sociodemographic details of the participants.

	Count	Proportion (in Percent)
Sex		
Men	18	21.4
Women	66	78.6
Diverse	0	0
	84	100
Qualification		
Doctor (IDW)	19	22.6
Doctor (OP)	15	17.9
Registered nurse (IDW)	14	16.7
Medical assistant (OP)	8	9.5
Medical students (IDW)	4	4.8
Medical students (OP)	3	3.5
Trainees (IDW)	1	1.2
Trainees (OP)	1	1.2
Other credentials	19	22.6
	84	100
Age		
16–25 years	14	16.7
26–35 years	26	30.9
36–45 years	15	17.9
46–55 years	18	21.4
>55 years	11	13.1
	84	100

**Table 2:** Key correlations.

Statement No.	1	2	3	4	5	8	9	12	15	20	28	29
1. It is important to pay attention to the amount of waste generated in everyday working life.	1											
2. I tell myself to act more sustainably than I really do.		1										
3. I can contribute to reducing the amount of waste myself.	0.323 **	−0.228 *	1									
4. I try to work in a way that saves resources	0.220 *			1								
5. I often find myself forced to act unsustainably or waste materials due to the stress/pressure of everyday life.		0.343 **		−0.258 *	1							
8. My knowledge of the correct disposal of waste is very good.		−0.230 *		0.220 *	−0.277 *	1						
9. My processes are already maximally efficient in terms of the utilization of resources.				0.276 *	−0.252 *	0.606 **	1					
12. The volume of waste is very high.					0.293 **			1				
15. I think it makes sense to introduce paper, plastic and residual waste separation in the patient’s/treatment room (…).	0.283 *	0.290 *				−0.241 *	−0.246 *		1			
20. I think it makes sense to introduce a wider range of sizes for dressing materials.					0.263 *					1		
28. I would like to have more guidelines and more help from the management levels to act sustainably.	0.222 *		0.269 *				−0.319 **		0.223 *	0.374 **	1	
29. I would like to see more guidelines and help from the management levels to work in a more resource-efficient and waste-reducing way.	0.261 *		0.247 *			−0.267 *	−0.333 **		0.249 *	0.363 **	0.962 **	1
* significant for *p* < 0.05 ** significant for *p* < 0.01	correlation coefficient (r)				r < −0.3	−0.3 < r < 0		0 < r < 0.3		0.3 < r < 0.6	r > 0.6	

## Data Availability

The dataset will be released on Zenodo (DOI: 10.5281/zenodo.17968537) once the manuscript is published.

## Supplementary Materials

The following supporting information can be downloaded at: https://doi.org/10.5281/zenodo.17968537, accessed on 31 March 2026, Video S1: Wieso unsere Erde eine Hauterkrankung hat und was Müll damit zu tun hat…, survey: questionnaire.

## Abbreviations

The following abbreviations are used in this manuscript:

CEA	Circular Economy Act
HCPs	Healthcare Professionals
IDW	Inpatient Dermatology Ward
OP	Outpatient Setting
WHO	World Health Organization
